# Supplementary material for: MetaRibo-Seq measures translation in microbiomes
Source: Nat Commun. 2020 Jun 29;11:3268. doi: 10.1038/s41467-020-17081-z (PMC7324362; doi:10.1038/s41467-020-17081-z)
Supplement: Supplementary file 10 — Supplementary Data 7 [file 41467_2020_17081_MOESM10_ESM.zip › File2/Confidence_VeryHigh_Taxonomy/378869_out.krona.html]

Javascript must be enabled to view this page.

members
magnitude
magnitudeUnassigned
count
unassigned
taxon
rank

378869\_out

13

2
superkingdom
13

201174
7
phylum

1760
class
7

85011
7
order

2062
7
family

7

SRS018936\_contig\_number\_contig-100\_2217.72569SRS019030\_contig\_number\_contig-100\_2859.2859SRS051882\_contig\_number\_20653SRS065176\_contig\_number\_contig-100\_1453.106591SRS1041138\_contig\_number\_2925SRS893358\_contig\_number\_11033SRS893378\_contig\_number\_11453
genus
1883

1239
6
phylum

4

SRS014683\_contig\_number\_18838SRS015217\_contig\_number\_contig-100\_275.185505SRS104084\_contig\_number\_contig-100\_284.87567SRS148874\_contig\_number\_contig-100\_130.97501
species
1262992

1

SRS077392\_contig\_number\_18553
species
1263007

186801
class
1

order
1
186802

species
1

SRS148721\_contig\_number\_20516
1950903
